# Human *SLC26A1* Gene Variants: A Pilot Study

**DOI:** 10.1155/2013/541710

**Published:** 2013-10-22

**Authors:** Paul A. Dawson, Pearl Sim, David W. Mudge, David Cowley

**Affiliations:** ^1^Mater Research, Translational Research Institute, Woolloongabba, QLD 4102, Australia; ^2^Department of Nephrology, University of Queensland at Princess Alexandra Hospital, Brisbane, QLD 4102, Australia; ^3^Pathology Department, Mater Misercordiae Hospital, South Brisbane, QLD 4101, Australia

## Abstract

Kidney stones are a global health problem, incurring massive health costs annually. Why stones recur in many patients remains unknown but likely involves environmental, physiological, and genetic factors. The solute linked carrier (SLC) 26A1 gene has previously been linked to kidney stones in mice. *SLC26A1* encodes the sulfate anion transporter 1 (SAT1) protein, and its loss in mice leads to hyperoxaluria and calcium oxalate renal stones. To investigate the possible involvement of SAT1 in human urolithiasis, we screened the *SLC26A1* gene in a cohort of 13 individuals with recurrent calcium oxalate urolithiasis, which is the commonest type. DNA sequence analyses showed missense mutations in seven patients: one individual was heterozygous R372H; 4 individuals were heterozygous Q556R; one patient was homozygous Q556R; and one patient with severe nephrocalcinosis (requiring nephrectomy) was homozygous Q556R and heterozygous M132T. The M132 amino acid in human SAT1 is conserved with 15 other species and is located within the third transmembrane domain of the predicted SAT1 protein structure, suggesting that this amino acid may be important for SAT1 function. These initial findings demonstrate genetic variants in *SLC26A1* of recurrent stone formers and warrant wider independent studies of *SLC26A1* in humans with recurrent calcium oxalate stones.

## 1. Introduction

The formation of insoluble calcium stones in the kidney and/or urinary tract is a major health problem worldwide with *≈*6–9% of men and *≈*3-4% of women affected [1]. This leads to significant cost in the form of expensive surgical procedures (>$2 billions annually in USA) and morbidity [2]. Most of these stones are composed of calcium and oxalate [3], the latter being a metabolic end product derived from the liver and diet [4], with high urinary oxalate levels (hyperoxaluria) being a major contributor to renal calcium oxalate deposition [5, 6]. We have previously shown normal levels of endogenous oxalate synthesis in recurrent stone formers [7] and individuals are generally compliant with dietary advice to reduce their oxalate intake, suggesting that most stones cannot be attributed to excess oxalate from the liver or diet. There is a genetic predisposition to renal stone formation with *≈*15% of patients having an affected family member and *≈*50% of patients form a second stone over the subsequent 10 years [8]. However, in most cases the aetiology of renal stone deposition is unknown [9]. 

Calcium oxalate stone formation is a multifactorial disease involving environmental, physiological, and genetic factors [12]. Numerous genetic studies have identified possible links between calcium oxalate stones and polymorphisms in genes that regulate calcium homeostasis [12–15], mediate inflammatory responses [16, 17], and maintain the structure and function of renal tubules [18–21]. Hyperoxaluria is the most frequent metabolic abnormality found in calcium oxalate stone formers and its basis can be idiopathic (most common), secondary (e.g., enteric hyperoxaluria due to intestinal dysfunction or resection), or due to primary hyperoxaluria (PH) [12]. The primary hyperoxalurias (PH 1 and PH 2) are rare forms of severe hyperoxaluria and calcium oxalate stones and are caused by mutations in the *AGXT* and *GRHPR* genes, respectively [22]. Monozygotic twin studies indicate that there is a genetic predisposition to idiopathic hyperoxaluria [12, 23], which may be relevant to the SAT1 sulfate anion transporter (protein is SAT1; gene is *SLC26A1*) that has been linked to calcium oxalate stone formation in mice [24].

SAT1 is expressed in the intestine, kidney, and liver where it exchanges sulfate (SO_4_^2−^) with structurally similar anions including oxalate (C_2_O_4_^2−^) [26–28]. This exchange process is proposed to be important for the removal of oxalate from the blood (via the intestine), as well as for maintaining circulating (via the kidneys) and hepatic levels of nutrient sulfate [4, 6, 24, 29–31]. Loss of SAT1 in mice led to disturbances in sulfate homeostasis (hyposulfataemia and hypersulfaturia), as well as hyperoxalaemia and hyperoxaluria [24]. The finding of calcium oxalate stones in SAT1 null mice in this landmark paper was remarkable because rodents are extremely refractive to forming stones, even when mild-moderate hyperoxaluria occurs [32].

To date, the contribution of human SAT1 to calcium oxalate stones is unknown. As an initial approach, we compared the *SLC26A1 *gene sequence of 13 male patients with recurrent calcium oxalate stones to NCBI reference *SLC26A1* sequences for human and 15 other species. Our findings of three nonsynonymous gene variants in *SLC26A1*, including a missense variant at a highly conserved residue in one patient with severe nephrocalcinosis, warrant wider independent studies of *SLC26A1* in recurrent calcium oxalate stone formers.

## 2. Materials and Methods

### 2.1. Patient Cohort

The research protocol was approved by the Mater Human Research Ethics Committee. All persons gave their informed consent prior to their inclusion in the study. The eligibility criteria for our study were adult patients with recurrent calcium oxalate stones, based on laboratory stone analysis (calcium oxalate >75% of the stone), for whom the aetiology of calcium oxalate stones was unknown, and if the diagnosis of primary hyperoxaluria or enteric hyperoxaluria was able to be excluded by their treating clinician. Buccal swab samples for DNA analysis were collected but individual patient characteristics were not.

### 2.2. Genomic DNA Isolation and PCR and DNA Sequencing

**Figure 1:**
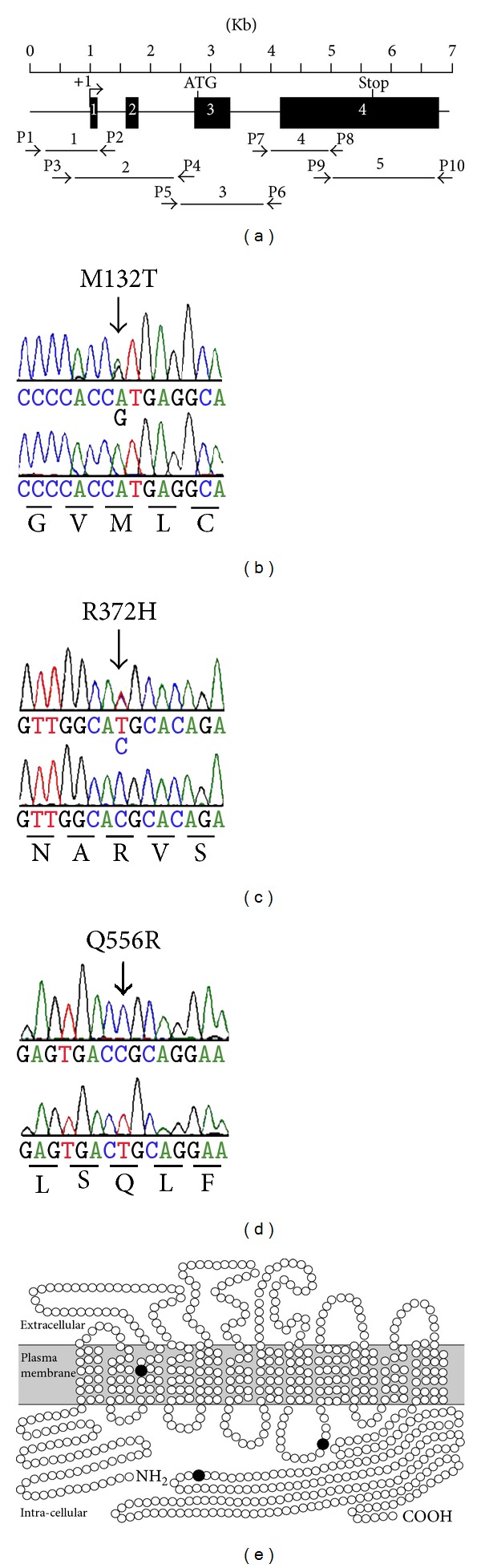
Identification of variants in the human *SLC26A1* gene and predicted amino acid substitutions. (a) Schematic of *SLC26A1* showing 4 exons (boxes), transcription initiation site (+1, arrow), and the relative location of PCR primers (P1–P10) and PCR amplicon fragments (1–5) used for DNA sequence analysis. ((b)–(d)) Representative DNA sequence chromatographs showing variant (*top panels*) and control (*bottom panels*) sequences that predict (b) heterozygous M132T, (c) heterozygous R372H, and (d) homozygous Q556R. Reverse complement nucleotide sequences are shown. (e) Position of amino acid substitutions (•) in the predicted secondary structure model of the human SAT1 protein [25]. M132T within the third transmembrane domain; R372H in the fourth intracellular loop; and Q556R in the intracellular carboxy-terminal domain.

Genomic DNA was prepared from buccal swabs according to the manufacturer's protocol (Isohelix). Five overlapping fragments of *SLC26A1* (Figure 1(a)) were PCR-amplified using 200 nM forward and reverse primers (Table 1) in a C1000 thermal cycler (BioRad). The thermal cycling protocol was 94°C for 1 min; 35 cycles of 94°C for 30 sec, 60°C for 30 sec, and 72°C for 2 min, followed by 1 cycle 72°C for 5 min. DNA sequencing of *SLC26A1 *was performed using 50 ng PCR product (purified with NucleoSpin Extract II, Macherey-Nagel) and 9.6 pmol forward and reverse primers (Table 1), ABI Prism BigDye Terminator Sequence chemistry and an AB3730x/96-capillary sequencer (Applied Biosystems). DNA sequence analyses included the *SLC26A1* coding region (2 exons of 2103 bp total), exon-intron junctions, 5′-(378 bp) and 3′-(1189 bp) UTRs and *≈*300 bp 5′-flanking region containing the minimal (135 bp) *SLC26A1* promoter region [33]. Nucleotide changes in *SLC26A1 *were verified by DNA sequence analysis of 2 independent PCR-amplified products for both sense and antisense strands.

### 2.3. Bioinformatic Analyses

Prediction of protein stability changes for amino acid substitutions was determined as previously described [10] using SAT1 amino acid sequence NP_998778.1. Alignment of SAT1 amino acid sequences between human (NP_998778.1) and multiple species was generated using the Clustal W program [11]. Putative transcription factor binding sites in the human *SLC26A1* 5′-flanking region were determined using MatInspector [34] and NCBI reference sequence NT_037622.5.

## 3. Results and Discussion

To date, two variants (G368S and Q556R) in the human *SLC26A1* gene have been reported, based on information from the NCBI single nucleotide polymorphism (SNP) database [25]. Whilst *SLC26A1* has yet to be associated with any human disease, the mouse *Slc26a1* gene has been linked to hyperoxaluria and calcium oxalate kidney stones [24]. Since human SAT1 and mouse Sat1 share high homology (78% amino acid identity) [33] and a similar tissue distribution with strong expression in the kidneys [27, 28], we propose a potential role for human *SLC26A1* in renal stone pathology.

**Figure 2:**
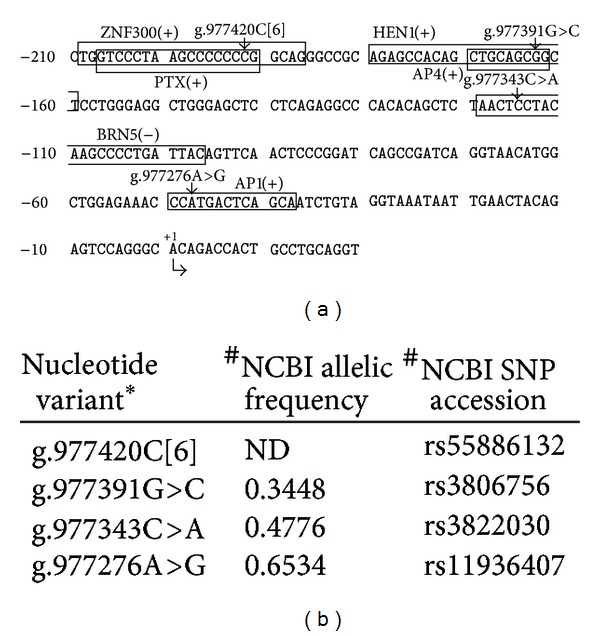
Sequence variants identified in this study and their location within putative transcription factor binding sites in the human *SLC26A1* 5′-flanking region. (a) Nucleotide sequence of the *SLC26A1* 5′-flanking region (NCBI accession number NT_037622.5) from −210 to +20 is shown. Position +1 denotes the putative transcription initiation site (NCBI accession number AK292747). Putative transcription factor binding motifs are boxed, and their position on the plus (+) or minus (−) strands is indicated: ZNF300: KRAB-containing zinc finger protein 300; PTX1: pituitary homeobox 1; HEN1: helix-loop-helix transcription factor; AP4: activator protein 4; BRN5: BM-5 POU domain factor; AP1: activator protein 1. (b) Allelic frequencies and accession numbers of nucleotide variants in the NCBI database: ^#^accessed 7 November 2012. *Nucleotide numbering based on NCBI reference sequence NT_037622.

In this study, we screened the *SLC26A1* gene in a cohort of recurrent calcium oxalate kidney stone formers and identified single nucleotide variants in the coding region of eight individuals (Table 2). Three of these variants predict amino acid substitutions (M132T, R372H, and Q556R), and four nucleotide variants are silent changes (T225T, S306S, R439R, and A625A). In addition, one variant was detected in the 5′-untranslated region of *SLC26A1 *exon 1 for three individuals, and four variants were found within the first 210 nucleotides of the *SLC26A1* 5′-flanking region for eight individuals (Table 2). The allele frequencies of 5 variants are shown in Table 3 and Figure 2. As yet, we do not know the allelic frequencies of *SLC26A1* variants in recurrent calcium oxalate stone formers but that will be the next phase of our work.

Destabilisation of protein structure is the most common mechanism by which an amino acid variant causes loss of protein function [35]. Using the Support Vector Machine program [10], we identified M132T and R372H to have a significant deleterious effect on the structural stability of human SAT1 protein (Table 3). In addition, the location of variants within membrane proteins can also be useful for predicting the functional consequences of amino acid changes. For example, nonconservative amino acid substitutions within the transmembrane domains (TMDs) of plasma membrane transporters frequently have a negative consequence on protein function [25]. The human SAT1 protein is predicted to contain 12 TMDs, with intracellular amino and carboxy-termini [28]. The nonconservative (hydrophobic to hydrophilic) M132T substitution is located within the third putative TMD of SAT1 (Figure 1(e)), suggesting that M132 may be important for maintaining the TMD structure of SAT1. R372H is located within the fourth intracellular loop, whereas Q556R is positioned in the intracellular carboxy-terminal sequence of the predicted secondary structure of SAT1 (Figure 1(e)), suggesting the possibility that these 2 amino acid substitutions may not disrupt the TMD structure of SAT1.

Conservation of amino acid sequences between multiple species can be useful to identify those residues that are important for protein function [36]. In order to determine if the nonsynonymous variants M132T, R372H, and Q556R occur at conserved positions of the SAT1 protein, we aligned the human SAT1 sequence with homologues from 15 different species (Table 4). Human M132 is conserved between all species, suggesting that this amino acid may play an important role in the structure and or function of SAT1. The R372H variant is a conservative amino acid change (i.e., both arginine and histidine are positively charged amino acids). Whilst human R372 is conserved with 11 other species (Table 4), this residue aligns with cysteine in rhesus monkey, serine in both rat and mouse, and glutamic acid in the African clawed frog (Table 4), implying that R372 may not be essential for SAT1 structure or function. Human Q556 aligns with an arginine residue in 10 species, including chimpanzee, rhesus monkey, dog, rat, and mouse, demonstrating that SAT1 sequence containing Q556R is conserved with other species. This finding implies that Q556 is a more recent amino acid in humans, due to genetic drift or selection, to become the major allele (*≈*0.65, Table 3). In addition, the presence of R556 in 10 species, together with the relatively high allelic frequency (*≈*0.35) of R556 in the human population (Table 3), suggests that the Q556R variant may not be pathogenetic in humans.

Previous studies identified the first 135 bp of the human *SLC26A1* 5′-flanking region to be sufficient for basal promoter activity [28]. In the *SLC26A1* 5′-flanking region of eight individuals, we identified four nucleotide variants (Table 2) which are located within putative transcription factor binding motifs (Figure 2(a)). One variant, g.977276A>G, is located within an AP1 site, previously shown to be important for the transcriptional activity of human *SLC26A1* [28]. However, this variant does not alter the essential core nucleotides of the AP1 binding motif sequence. Similarly, the other three variants found within the 5′-flanking region do not alter the essential core nucleotide sequences of the ZNF300, HEN1, or BRN5 transcription factor binding motifs, indicating that these variants may not be detrimental to the transcriptional activity of the *SLC26A1* promoter. These findings, together with the high allelic frequencies (0.34 to 0.65, NCBI database) of g.977391G>C, g.977343C>A, and g.977276A>G in the general population (Figure 2(b)), imply that these variants may not be pathogenetic.

## 4. Conclusions

This study provides preliminary and as yet unreported evidence of sequence variants in the human *SLC26A1* gene of a small number of individuals with recurrent calcium oxalate kidney stones. Of great interest is the finding of a nonconservative amino acid substitution (M132T) within a predicted transmembrane domain of SAT1, in one patient with severe nephrocalcinosis. The novelty of these findings warrants further studies of *SLC26A1* in humans with unexplained recurrent kidney stones.

## Figures and Tables

**Table 1:** Primers used for PCR and DNA sequencing.

Primer	^ a^Direction	Sequence (5′ to 3′)	^ b^PCR fragment
Primers used for PCR			
P1	F	GCTGAGGCAGGAGAATGGCGTGAAC	1
P2	R	CTGGGGTCCAGGTGTGTGGAATGG	1
P3	F	CAGCTTGGACCAGGCTGGCTCCTTG	2
P4	R	GGATTGGTGCTGGGCCTTCTCCT	2
P5	F	GTAATCCCATCTCACCTCACGATG	3
P6	R	CATCGAGCAGTGGCTGTGAGAGGTAG	3
P7	F	GATGCTCACGTGGATGTCACAGTTG	4
P8	R	CGAACTCTGTGGCATCCTCGTAGAAG	4
P9	F	CACCTGTATGCTGGTCAGCACAGAG	5
P10	R	TGTCCCATTCCTTCCACCTAGAG	5
Primers used for sequencing			
P11	F	CCAGGAGGCAGAGCTTCCAG	1
P12	F	GCATATGCACGCACCGGCAGCCTTG	1
P13	R	AGCCTGAGGGTGCCCCTAAGAAACC	1
P14	R	CAAGGAGCCAGCCTGGTCCAAGCTG	1
P15	F	CTGGCTCCTTG CAGGACCAG	2
P16	F	CCATTCCACACACCTGGACCCCAG	2
P17	R	CTTCTCAGCGACGTTAGGCAAAGAC	2
P18	R	CATCGTGAGGTGAGATGGGATTAC	2
P19	F	AGGAGAAGGCCCAGCACCAATCC	3
P20	F	CAAGCACAGGGTTGGCAGAGGAGGTG	3
P21	R	CCTCACCAGCACCTGGCATGG	3
P22	R	TGCCCAAGCCTTGCTGTCTTG	3
P23	F	TTGTCAGGTCCTGTGCCGTGAC	4
P24	R	CTCTGTGCTGACCAGCATACAGGTG	4
P25	F	CTTCTACGAGGATGCCACAGAGTTCG	5
P25	F	ATCACACGCAGGACCCAAACACTCAG	5
P26	R	ATTCCTTCCACCTAGAGCTGAGG	5
P27	R	CTGAGTGTTTGGGTCCTGCGTGTGAT	5

^a^F: forward primer; R: reverse primer. ^b^PCR fragment denoted in Figure 1(a).

**Table 2:** *SLC26A1* sequence variants identified in a cohort of recurrent calcium oxalate stone formers.

	^ a^Individuals with recurrent calcium oxalate kidney stones												
Sequence variant	A	B	C	^ b^D	E	F	G	H	I	J	K	L	M
*Coding nonsynonymous *													
c.395T>C, p.M132T	−	−	−	+	−	−	−	−	−	−	−	−	−
c.1115G>A, p.R372H	−	−	−	−	−	−	−	−	−	−	−	+	−
c.1667A>G, p.Q556R	−	++	−	++	+	+	−	−	−	−	+	−	+
*Coding synonymous *													
c.675C>A, p.T225T	−	−	−	−	−	+	−	−	−	−	−	−	+
c.918G>A, p.S306S	−	+	−	−	+	+	+	−	−	−	+	−	+
c.1315C>A, p.R439R	−	−	−	+	−	−	−	−	−	−	−	−	−
c.1875C>T, p.A625A	−	+	−	−	+	+	+	−	−	−	+	−	+
5′* Untranslated region* ^c^													
g.977205C>T	+	−	+	−	−	−	+	−	−	−	−	+	−
5′* Flanking region* ^c^													
g.977276A>G	−	+	+	++	+	−	+	+	+	−	+	−	−
g.977343C>A	−	+	−	+	+	−	+	+	+	−	+	−	−
g.977391G>C	−	−	−	−	+	−	+	+	−	−	+	−	−
g.977420C[6]	−	−	−	−	−	−	+	−	−	−	−	−	−

^a^The genotypes of thirteen individuals (A–M) are indicated as + (*heterozygous*) or ++ (*homozygous*) for each sequence variant. ^b^Individual (D) with severe nephrocalcinosis requiring left nephrectomy. ^c^Genomic DNA reference NT_037622.5.

**Table 3:** Predicted effect of missense gene variants on SAT1 protein structure stability, and allelic frequencies of these variants in the NCBI SNP database.

*SLC26A1* variant	^ a^Stability prediction	^ b^SVM score	Allelic frequency this study	Allelic frequency ^c^NCBI database	^ c^NCBI SNP accession
M132T	decrease	−0.9	0.038	—	—
R372H	decrease	−1.0	0.038	0.0082	rs73219719
Q556R	increase	0.2	0.307	0.3484	rs3796622

^a^Prediction of protein stability and ^b^Support Vector Machine modelling scores (values less than zero are significant) [10]. ^c^NCBI database accessed on October 30 2012.

**Table 4:** Alignment of human SAT1 amino acid variants across species.

Species, accession number	% Identity to human SAT1	Human SAT1 variants		
		^ a^M132T	R372H	Q556R
Human	100	M	R	Q
Chimpanzee, XM_003310211.1	98	M	R	R
Pygmy chimpanzee, XM_003811300.1	98	M	R	R
Sumatran orangutan, XM_002814495.1	96	M	R	R
Northern white-cheeked gibbon, XM_003280551.1	96	M	R	R
Olive baboon, XM_003890923.1	95	M	R	R
Rhesus monkey, XM_001093208.2	94	M	C	R
Bolivian squirrel monkey, XM_003934620.1	93	M	R	R
Dog, XM_545984.4	80	M	R	R
Cattle, XM_002688445.1	79	M	R	Q
Rat, CH474079.2	78	M	S	R
Mouse, BC025824.1	78	M	S	R
African clawed frog, NM_001090973.1	51	M	E	K
Rainbow trout, NM_001124486.1	50	M	R	K
Japanese eel, AB111927.1	49	M	R	K
Zebra fish, NM_001080667.1	48	M	R	K

Human SAT1 variants detected in this study are shown at the top, and the aligned amino acid for each species is shown below. Alignments were generated using the Clustal W program [11]. ^a^Identical amino acid across species.
